# Application of VAP bundles resulting in low incidence of VAP in ICU

**DOI:** 10.1186/1753-6561-5-S6-P68

**Published:** 2011-06-29

**Authors:** FM Khan, A Bokhamsin, J Carol

**Affiliations:** 1 Prevention and Control of Infection, Mouwasat Hospitals Damman Saudi Arabia, Dammam, Saudi Arabia

## Introduction / objectives

Ventilator-associated pneumonia (VAP) is an airways infection that must have developed more than 48 hours after the patient was intubated. Reducing mortality due to ventilator-associated pneumonia requires an organized process that guarantees early recognition of pneumonia and consistent application of the best evidence-based practices. The Ventilator Bundle is a series of interventions developed by IHI related to ventilator care that, when implemented together, will achieve significantly better outcomes than when implemented individually.

## Methods

This study was conducted in our 24 bedded Adult Medical Surgical ICU. VAP Bundle Program was implemented by our multidisciplinary Team, the VAP Bundle Team and implemented in July 2009. Surveillance reports from ICU for the year 2009-2010 were reviewed. Data collected and analyzed for ventilated-associated pneumonia (VAP) for the same period of time and compared before and after intervention.

## Results

There were a total of 5612 patients admitted to ICU in 2010 and out of that 345 patients required mechincal ventilation for the year 2010. The data was analyzed and compared on quarterly basis based on 100 ventilator days. Fifteen patients developed VAP during the above period. The results shows a gradual decline in the VAP rate towards the end of the 4th quarter in 2010. The results clearly shows the difference between pre and post-intervention period and lower VAP rate in 2010.

## Conclusion

VAP is a patient safety concern that can be prevented with evidence-based interventions. Lessening VAP rates will shorten hospitalization and reduce morbidity, saving lives as well as money. Many hospitals have since implemented the Ventilator Bundle in their ICUs and have reported significant decreases in VAPs or long periods of time (one year or longer) with no VAPs in their patients.

## Disclosure of interest

None declared.

